# Treatment terminations during radiation therapy: A retrospective descriptive single-center analysis

**DOI:** 10.1371/journal.pone.0350496

**Published:** 2026-06-04

**Authors:** Alaattin Ozen, Ilknur Harmankaya, Canan Ozdemır, Mehmet Halıcı, Oya Coskun, Ekin Baran Guler, Ozge Atılla, Sumeyra Can

**Affiliations:** Basaksehir Cam and Sakura City Hospital, Division of Radiation Oncology, Istanbul, Turkiye; All India Institute of Medical Sciences, INDIA

## Abstract

**Background:**

Radiotherapy (RT) is a cornerstone of cancer management, substantially improving local tumor control and overall survival. However, a subset of patients fail to complete the prescribed RT course. Identifying the factors associated with treatment termination is essential to enhancing cancer care delivery and patient outcomes.

**Methods:**

This retrospective, single-center analysis included 10039 patients who underwent RT between January 2020 and December 2024. Patients who terminated treatment before completion were identified in institutional RT records. Demographic and clinical characteristics, treatment intent, and reasons for termination were primarily evaluated using descriptive statistical analyses, with exploratory comparative analyses performed between curative- and palliative-intent groups.

**Results:**

Between 01/01/2020 and 12/31/2024, RT was terminated in 297/10039 patients (2.96%). The most leading causes of termination was deterioration in performance status (143 patients, 48.1%). Of these, 170 patients (57.2%) had been treated with palliative intent. Lung cancer (96 patients, 32.3%) was the most frequent primary diagnosis, while the brain (92 patients, 31%) was the most commonly irradiated site. The median number of prescribed fractions was 13 (range: 2–44), and patients completed a median of 51.6% (range: 5–93%) of these fractions before termination. The most common reason was deterioration in performance status (48.1%). Treatment termination rates were significantly higher in palliative cases compared with curative cases (6.13% [170/2,772] vs. 1.75% [127/7,267]; χ² = 134.4; p < 0.001). Relative risk analysis indicated that palliative-intent patients had a 3.50-fold higher risk of treatment termination. Performance deterioration was more frequent in the palliative group (72.4% vs. 27.3%; p < 0.001). Treatment-related toxicity (grade III-IV) occurred predominantly in curative-intent patients (88.9% vs. 11.1%; p < 0.001).

**Conclusion:**

Most RT terminations occurred among patients with poor performance status and advanced disease. These findings suggest that multidisciplinary supportive care may be relevant and should be evaluated in future prospective studies.

## Introduction

Radiotherapy (RT) may be delivered with curative or palliative intent and can utilize conformal, intensity-modulated, or stereotactic techniques. It may be administered alone or alongside systemic cancer treatments. Approximately 50–60% of cancer patients require RT at some point in their disease course [1,2]. Although institutional rates vary, palliative RT comprises 30–50% of all radiotherapy practices [3].

Because the biological effectiveness of RT depends on uninterrupted delivery, completing the prescribed treatment plan is essential. However, a proportion of patients fail to complete RT due to medical, logistical, or psychosocial reasons [4–6]. Patients treated with palliative intent, who often have poor baseline performance status and advanced disease, are especially vulnerable to early treatment termination [6–9].

Common contributors to RT termination include acute toxicities from RT or systemic therapy, performance deterioration, comorbid illnesses, infections such as COVID-19, transportation difficulties, and patient preference or withdrawal [5,7,8].

Premature termination compromises intended therapeutic benefit and may lead to avoidable toxicity, reduced quality of life, inefficient resource utilization, and financial burden. Identifying the incidence and underlying causes of RT termination is therefore essential, particularly in modern, value-based oncology care models [9–11].

This retrospective descriptive study aimed to characterize the frequency and documented reasons for radiotherapy (RT) discontinuation and to describe associated clinical and demographic features according to treatment intent.

## Materials and methods

### General study details

The study was designed as a descriptive retrospective analysis and was not intended to evaluate causal relationships or independent predictors of treatment termination. This retrospective, single-center study included adult patients (≥18 years) who were scheduled to receive radiotherapy (RT) between 01/01/2020 and 12/31/2024. The study was approved by the Institutional Ethics Committee of City Hospital (Approval No: 2025-175; Date: May 21, 2025; KAEK/21.05.175). The data were accessed for research purposes on 01/06/2025. Due to its retrospective design, the requirement for written informed consent was waived. The study adhered to the principles of the Declaration of Helsinki and Good Clinical Practice (GCP) guidelines. No external financial support was received.

### Participants

Inclusion criteria were: age ≥ 18 years, histologically confirmed malignancy, completion of CT-based RT simulation with consent, and termination of the prescribed radiotherapy course after the delivery of at least one treatment fraction. The patients who failed to initiate RT after simulation were excluded from the analysis. Exclusion criteria also included insufficient clinical documentation. Patients who did not start radiotherapy after the simulation for any reason were not included in the study.

Clinical and demographic data were retrospectively retrieved from electronic medical records, including age, sex, primary tumor site, treatment intent (curative/palliative), and the documented reason for treatment termination. Radiotherapy termination was defined as termination of the prescribed RT course after the administration of at least one treatment fraction, resulting in termination of the planned treatment schedule. Reasons for termination were categorized as decline in performance status, patient preference, death, grade III-IV treatment-related toxicity (RT, chemotherapy, or both), COVID-19 infection, or change in treatment decision (for a reason other than the primary treatment purpose; surgery, systemic therapy, or best supportive care).

Baseline and follow-up performance status scores, such as the Eastern Cooperative Oncology Group (ECOG) or Karnofsky Performance Status (KPS), were not consistently documented in the electronic medical records and were therefore not available for analysis. Consequently, deterioration in performance status was determined based on physician-documented clinical assessments recorded at the time of treatment termination.

### Statistical analysis

All statistical analyses were performed using IBM SPSS Statistics for Windows, Version 20.0 (IBM Corp., Armonk, NY, USA). Because the primary objective of the study was descriptive, analyses focused mainly on summarizing patient characteristics, treatment features, and documented reasons for radiotherapy termination. Comparative statistical tests between curative- and palliative-intent groups were exploratory in nature and intended to identify potential associations rather than establish causality. Continuous variables were expressed as means ± standard deviations or medians with interquartile ranges (IQR), depending on the normality of distribution assessed by the Shapiro–Wilk test [Shapiro & Wilk, 1965]. Categorical variables were summarized as frequencies and percentages. Comparisons between groups were made using the Chi-square test or Fisher’s exact test for categorical variables, and the Independent Samples t-test or Mann–Whitney U test for continuous variables, as appropriate. Univariate analyses were first performed to identify variables associated with treatment termination. A p-value <0.05 was considered statistically significant. Given the descriptive nature of the study, multivariable regression analyses were not performed.

## Results

**Fig 1:**
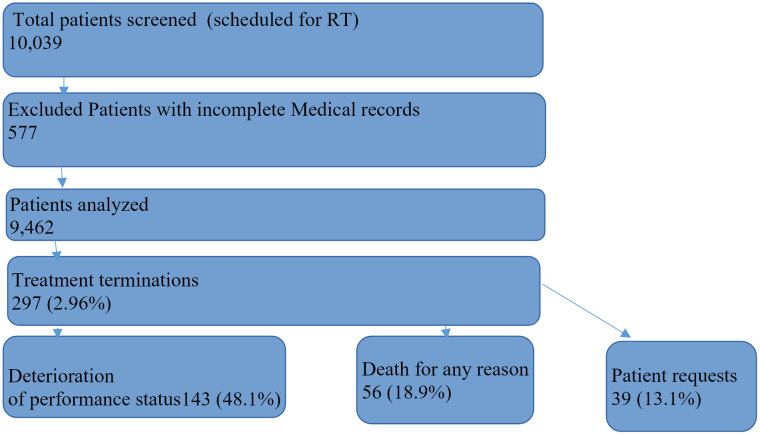
Consort diagram.

Between 01/01/2020 and 12/31/2024, a total of 10,039 patients were scheduled for radiotherapy (RT) and underwent treatment planning. Among them, 297/10039 patients (2.96%) terminated RT after receiving at least one treatment fraction. A schematic patient flow is depicted in Fig 1.

The median age was 64 years (range: 27–93), and 62.6% of the cohort were male (Table 1). The most common primary tumor sites were lung (n = 96, 32.3%), gastrointestinal system (n = 65, 21.9%), and brain (n = 33, 11.1%). Most patients presented with non-metastatic disease (n = 177, 59.6%), while 120 patients (40.4%) had metastatic involvement. By treatment intent, 170 patients (57.2%) were scheduled for palliative RT, whereas 127 (42.8%) were planned for curative treatment.

**Table 1:** Patients’ characteristics.

Parameters	Value
**Age** Median (range)	64 (27-93)
**Sex (n, %)** Male Female	186 (62.6) 111 (37.4)
**Primary diagnosis (n, %)** Lung cancer Gastrointestinal cancer Brain cancer Urinary system cancer Gynecological cancer Breast cancer Head and neck cancer Hematological cancer Primary unknown cancer Skin cancer Soft Tissue Sarcomas Bone cancer	96 (32.3) 65 (21.9) 33 (11.1) 31 (10.4) 23 (7.7) 13 (4.4) 12 (4.0) 6 (2.0) 6 (2.0) 5 (1.7) 5 (1.7) 2 (0.7)
**Stage (n, %)** Non-metastatic Metastatic	177 (59.6) 120 (40.4)
**Treatment aim (n, %)** Palliative Curative	170 (57.2) 127 (42.8)
**Irradiated body area (n, %)** Brain Bone Thorax Pelvic Upper abdomen Head and neck Inguinal Axillary Breast	92 (31.0) 59 (19.8) 58 (19.5) 52 (17.5) 27 (9.1) 5 (1.6) 2 (0.7) 1 (0.3) 1 (0.3)
**RT plan** 3D-CRT/IMRT SRS/SBRT	292 (98.3) 5 (1.7)
**Planned RT fraction** Median (range)	13 (2-44)
**RT completion rate before termination (%)** Median (range)	51.6 (5-93)

Abbreviations: RT = Radiotherapy; 3D-CRT = Three-Dimensional Conformal Radiotherapy; IMRT = Intensity-Modulated Radiotherapy; SRS = Stereotactic Radiosurgery; SBRT = Stereotactic Body Radiotherapy.

The most frequently treated anatomical regions were the brain (31.0%), bone (19.8%), thorax (19.5%), and pelvic area (17.5%). Only 1.7% (n = 5) of patients received stereotactic radiosurgery or stereotactic body RT (SRS/SBRT). The median number of prescribed fractions was 13 (range: 2–44), and patients completed a median of 51.6% (range: 5–93%) of these fractions before termination.

**Fig 2:**
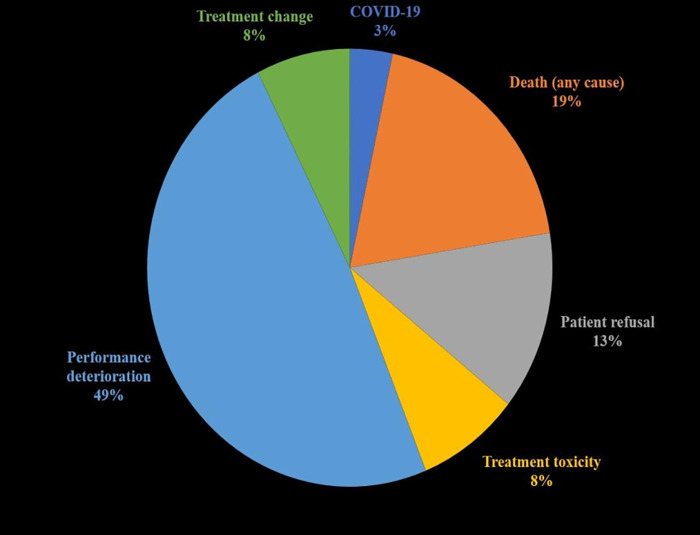
Distribution of RT termination causes in the entire cohort.

The distribution of termination causes is shown in Fig 2. The most common reason was deterioration in performance status (48.1%), followed by death (18.9%) and patient preference (13.1%). Treatment termination rates were significantly higher in palliative cases compared with curative cases (6.13% [170/2,772] vs. 1.75% [127/7,267]; χ² = 134.4; p < 0.001), indicating a strong association between treatment intent and RT termination. Relative risk analysis indicated that palliative-intent patients had a 3.50-fold higher risk of treatment termination.

**Fig 3:**
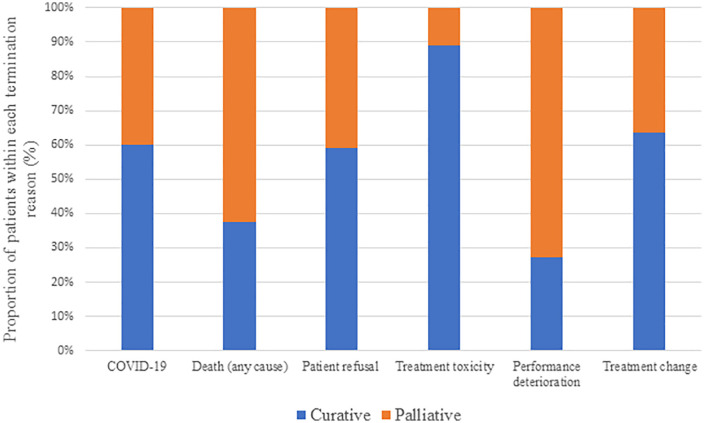
Proportion of patients within each termination reason (curative vs. palliative).

Table 2 and Fig 3 summarize reasons for termination according to treatment intent. Performance deterioration was more frequent in the palliative group (72.4% vs. 27.3%; p < 0.001). Treatment-related toxicity (grade III-IV) occurred predominantly in curative-intent patients (88.9% vs. 11.1%; p < 0.001). Additionally, patient preference and treatment-decision changes were more common in curative cases (p = 0.028 and p = 0.040, respectively).

**Table 2:** Distribution of radiotherapy termination reasons in curative vs. palliative patients.

Reason for RT termination	Curative %* (n)	Palliative %* (n)	p-value**
COVID-19 (n = 10)	60.0 (6)	40.0 (4)	0.262
Death (any cause) (n = 56)	37.5 (21)	62.5 (35)	0.377
Patient preference (n = 39)	59.0 (23)	41.0 (16)	**0.028**
Treatment toxicity (grade III-IV) (n = 27)	88.9 (24)	11.1 (3)	**<0.001**
Performance deterioration (n = 143)	27.3 (39)	72.7 (104)	**<0.001**
Treatment change (n = 22)	63.6 (14)	36.4 (8)	**0.040**

*Percentages represent the distribution of treatment intent within each reason for RT termination, **Comparative analysis using the chi-square test, p-values obtained from separate 2 × 2 comparisons for each reason

There were no significant differences between groups regarding COVID-19–related terminations (p = 0.262) or death (p = 0.377). A greater proportion of palliative patients completed more than half of their planned fractions (60.0% vs. 43.3%; p = 0.004).

The median number of planned fractions was higher in the curative group (28 vs. 10), yet their completion rate was lower (39% vs. 60%; Table 3). Overall, the proportion of prescribed fractions delivered was significantly higher among patients receiving palliative RT (p = 0.008).

**Table 3:** Comparison of treatment duration and completion rates.

	Curative (n = 127)	Palliative (n = 170)	p-value
**Number of planned total fractions**, **Median (range)**	28 (5-44)	10 (2-18)	< 0.001*
**RT completion rate before termination,** **Median (range)**	39 (5-93)	60 (6-90)	0.008*
**RT completion ≥ 50%,** **n (% of group)**	55 (43.3%)	102 (60.0%)	0.004**

*Mann–Whitney U, **Chi-square tests.

## Discussion

This study focused exclusively on patients who terminated radiotherapy after treatment initiation. Patients who failed to start RT following simulation were not included, as the determinants of non-initiation may differ substantially from those associated with treatment termination. In our cohort, the RT termination rate was 2.96%, consistent with recently reported contemporary rates ranging from 2–5% in modern radiotherapy practice [9,12]. Deterioration in performance status remained the leading reason for early termination, aligning with multiple recent studies—especially those involving patients with brain metastases receiving palliative RT [1,13].

Performance decline during RT is particularly important, as recent evidence demonstrates that patients whose ECOG status worsens during palliative RT have dramatically reduced survival durations—often measured in weeks rather than months [13,14]. In this context, our findings showing significantly higher termination rate among palliative-intent patients are in agreement with large multi-center studies and national database analyses [6,10,15]. Although patients treated with palliative intent exhibited higher rates of radiotherapy termination, they paradoxically completed a greater proportion of their prescribed fractions compared with those receiving curative treatment. This apparent contradiction is most likely attributable to differences in fractionation schedules. Palliative radiotherapy typically employs shorter, hypofractionated regimens designed to provide rapid symptom relief and accommodate the limited life expectancy and performance status of these patients. Consequently, even when treatment is terminated, patients are more likely to have completed a substantial proportion of the planned course. In contrast, curative-intent treatments generally involve longer fractionation schedules, making them more susceptible to termination from acute toxicities, intercurrent illnesses, or logistical challenges before a comparable proportion of treatment is delivered. This observation may be explained by our data demonstrating a significantly shorter median number of planned fractions in the palliative group compared with the curative group (10 vs. 28).

Treatment-related toxicity was more commonly a cause for termination among curative-intent patients, which is consistent with findings showing increased risk of acute toxicity in longer-course radical RT regimens, especially when given with concurrent chemotherapy [4,5,15].

Sociodemographic and logistical barriers—including comorbidity burden, socioeconomic status, insurance limitations, and transportation challenges—have been shown in national datasets to significantly influence early RT termination [10,15]. Although our study lacked these variables, the patterns observed are broadly aligned with those reported in larger population-level analyses.

COVID-19–related treatment termination in our cohort remained low, supporting the global findings that modified workflow protocols and increased use of hypofractionation successfully mitigated pandemic-associated risks and treatment delays [8,16].

This study has several limitations that should be acknowledged. First, its single-center design may limit the generalizability of the findings to other institutions with different patient populations, healthcare resources, and clinical workflows. Institutional practice patterns—such as referral pathways, patient selection criteria, availability of multidisciplinary supportive care, and preferences for fractionation or treatment intent—can significantly influence radiotherapy utilization and termination rates. Additionally, the study was conducted at a high-volume tertiary cancer center, where access to advanced technologies and specialized supportive services may not reflect the conditions of smaller or resource-limited settings. Therefore, caution should be exercised when extrapolating these results to broader populations. Future multicenter and prospective studies are warranted to validate these findings and enhance their external applicability.

The higher observed RT termination rate among patients receiving palliative treatment suggests the vulnerability of this population and highlights the importance of early risk assessment, individualized supportive care, and flexible adaptation of therapy. A patient-centered clinical approach—including multidisciplinary evaluation, symptom management, and ongoing performance monitoring—may enhance treatment adherence and overall outcomes [13–18].

As this was a descriptive retrospective study, the findings should be interpreted as hypothesis-generating. The observed patterns suggest that multidisciplinary supportive care may warrant further prospective evaluation.

## Conclusion

Radiotherapy termination occurred in a minority of patients, most commonly due to deterioration in performance status, and was more frequent among those receiving palliative-intent treatment. Despite higher termination rates, palliative patients completed a greater proportion of their prescribed fractions, likely reflecting the shorter hypofractionated schedules used in this setting. The potential role of multidisciplinary supportive care in improving treatment adherence was not directly evaluated and should therefore be considered hypothesis-generating. Given the retrospective, single-center design, caution is required when generalizing these findings. Prospective multicenter studies are warranted to confirm these observations and to explore strategies for minimizing avoidable treatment termination.
